# Greenness and pollution exposure predict corticosterone concentration in an urban songbird

**DOI:** 10.3389/fphys.2025.1603811

**Published:** 2025-06-25

**Authors:** Mikus Abolins-Abols, Ray Yeager, Jay Turner, Ted Smith, Aruni Bhatnagar

**Affiliations:** ^1^ Department of Biology, University of Louisville, Louisville, KY, United States; ^2^ Center for Integrative Environmental Health Sciences, University of Louisville, Louisville, KY, United States; ^3^ Christina Lee Brown Envirome Institute, University of Louisville, Louisville, KY, United States; ^4^ Division of Environmental Medicine, Department of Medicine, University of Louisville, Louisville, KY, United States; ^5^ Superfund Research Center, University of Louisville, Louisville, KY, United States; ^6^ Department of Energy, Environmental and Chemical Engineering, Washington University in St. Louis, St. Louis, MO, United States

**Keywords:** greenness, air pollution, urban, ecology, avian physiology

## Abstract

Air pollution is known to negatively affect avian health, and some air pollutants have been suggested to drive changes in bird population size at a regional level. Although several studies have investigated the effect of air pollution on bird health, how air pollution exposure is associated with avian physiology at a local scale is not known. Moreover, the extent to which avian health may be affected by vegetation, which modulates pollutant deposition and dispersion, has not been assessed. Here we combine high-resolution mapping of major air pollutants (NO_2_ and ultrafine particles) and vegetation types with dense spatial sampling of American robins, an urban exploiter, to ask how air pollution exposure, vegetation, and their interaction predict baseline corticosterone and bird condition. The relationships between environmental variables and physiological metrics were assessed at various distances from the capture location. We found that elevated air NO_2_ concentration is associated with higher baseline corticosterone levels within 500 m of the capture location. Vegetation did not modulate the relationship between corticosterone and NO_2_. We found sex-dependent relationships between greenness, corticosterone, and body weight. Within 20 m from the capture locations female corticosterone showed negative relationship with leaf area index, while female body weight was positivity related to the overall greenness. These relationships were absent in males. Collectively, the results of this study show that variations in air pollution and vegetation at a local intra-neighborhood scale predict fitness- and stress-related markers in an urban songbird.

## Introduction

Exposure to air pollution is a critical global environmental health issue with documented adverse health effects in both human and non-human animals, including birds (Newman, 1979; Llacuna et al., 1996; Sanderfoot and Holloway, 2017; Landrigan et al., 2018). In human populations, air pollution exposure is associated with 4.5 million premature deaths per year (Landrigan et al., 2018). Air pollution is estimated to affect wildlife population sizes as well. For instance, reduction in ozone due to U.S. EPA NOx Budget Trading Program is estimated to have averted the loss of 1.5 billion birds over the last 50 years, with the strongest effect seen in small land birds (Liang et al., 2020). However, the mechanisms by which air pollution affects bird health, and the environmental factors that may mediate this relationship, are not well understood (Barton et al., 2023).

Birds have very efficient respiratory systems, characterized by unidirectional air flow and countercurrent gas exchange, which makes their respiration more susceptible to the adverse effects of particulate matter and gaseous pollution (Sanderfoot and Holloway, 2017). Although most studies investigating the effect of air pollutants on avian health show a negative effect on one or more fitness-relevant traits, important knowledge gaps persist (Barton et al., 2023). These gaps include lack of understanding of how different species respond to pollution (Salmón et al., 2018), lack of studies on particular pollutants (e.g., nitrogen dioxide), and lack of studies investigating the effect of pollution-mediating environmental factors on avian health (Barton et al., 2023).

Among the most significant air pollutants are those generated by the combustion of fossil fuels. These include nitrogen and sulfur oxides, particulate matter, volatile organic compounds (VOCs), and heavy metals (Sanderfoot and Holloway, 2017). These air pollutants can damage respiratory tissues (Rombout et al., 1991), cause oxidative stress (Herrera-Dueñas et al., 2014; Isaksson et al., 2017; Salmón et al., 2018), are associated with lower body condition (Wemer et al., 2021), and can decrease avian reproductive output (reviewed in Sanderfoot and Holloway, 2017; Barton et al., 2023). A commonly observed physiological response to pollution exposure in animals is the elevation of corticosterone levels.

Corticosterone is glucocorticoid hormone that mediates metabolism and the physiological stress-response (Romero and Butler, 2007). Corticosterone and other glucocorticoids play a number of different roles in the stress response, including preparing the organism to respond to stressors, enhancing the response to stressors, and facilitating recovery from stressors (reviewed in Sapolsky et al., 2000). In rodents, exposure to particulate matter and ozone activates stress-responsive brain regions (Sirivelu et al., 2006; Gackière et al., 2011) and increases corticosterone levels (Sirivelu et al., 2006; Martrette et al., 2011). In birds, experimental exposure to a mix VOCs and oxidizing gases (SO_2_, NO_2_) resulted in an increase in corticosterone levels in kestrels and quail (Cruz-Martinez et al., 2015). Wild tree swallow nestlings at various distances from oil sands extraction sites with different NO_2_ and VOC levels, however, showed no difference in corticosterone levels. Corticosterone levels have also been shown to differ across a heavy metal pollution gradient: baseline corticosterone levels have been positively associated with blood lead levels in house sparrows across an urban-rural gradient (White et al., 2022), although other studies show no relationship between corticosterone and metals pollution (Li et al., 2021). Both baseline and stress-induced corticosterone levels have also been shown to differ across a more general urban-rural gradient, although the directionality of the difference varies across species, with some species showing higher corticosterone levels in urban compared to rural areas, while others show lower or no difference in corticosterone levels in urban compared to rural areas (Deviche et al., 2023). Focusing on investigating relationships between specific stressors and corticosterone may help bring clarity to these differences.

Most field studies investigating the link between pollution and avian health measure physiological metrics and variation in pollution across a coarse urban-rural gradient. These field studies show that a strong urban-rural spatial gradient in air pollution is associated with increased oxidative stress (Herrera-Dueñas et al., 2014; Isaksson et al., 2017; Salmón et al., 2018), increased heterophil/lymphocyte ratio (Bauerová et al., 2017), elevated corticosterone (White et al., 2022), increased inflammation (Isaksson et al., 2017), shorter telomeres (Salmón et al., 2016), lower body mass (Wemer et al., 2021), and increased hemopoiesis (Li et al., 2021). However, air pollution from roadways dissipates within a couple hundred meters of the road (Baldwin et al., 2015), which can create a strong spatial heterogeneity in pollution within local urban environments. To our knowledge, it is not known whether fine-scale variation in air pollution within urban areas is associated with differences in avian health.

A contributing factor to the heterogeneity in pollution exposure in urban areas is vegetation. Vegetation can affect both the dispersal and deposition rates of air pollutants (Janhäll, 2015; Tiwari et al., 2019). The large surface area of plant leaves may facilitate increased deposition of both gaseous and particulate pollutants (Janhäll, 2015). Vegetation can also influence the movement of air and, as a consequence, the dispersion of air pollutants (Xing and Brimblecombe, 2019). The interaction between vegetation and air pollution is complex because, depending on the type of vegetation and its structure, green urban infrastructure can either decrease or increase pollutant exposure (Janhäll, 2015; Xing and Brimblecombe, 2019). To our knowledge, no study has investigated how vegetation and pollution exposure interact to mediate avian health (Barton et al., 2023).

Vegetation plays other fundamental roles in avian urban ecology in addition to its role in mediating pollutant exposure. Vegetation provides habitat for birds and modulates thermal microenvironment by providing shade (Armson et al., 2012). Vegetation also serves as a food source for frugivorous urban birds and a habitat for insectivorous bird prey. The amount and structure of vegetation may therefore affect avian health by both pollution-dependent and independent mechanisms.

Importantly, urban green infrastructure contains different types of vegetation. Grass and tree canopy, for example, may have different effects on pollution dispersal and deposition (Janhäll, 2015). These two major types of vegetation also provide different habitat and food resources for birds, differ in their ability to modulate temperature (Armson et al., 2012), and may harbor different disease vectors. Partitioning urban vegetation across different types is thus important to assess the effect of urban green infrastructure on bird health. While studies have investigated the association between different vegetation types and bird abundance (Blair, 2004), to our knowledge, no study to date has investigate how different types of vegetation are associated with physiological markers of stress and health.

In this paper we investigated the relationship between two major urban air pollutants, three urban vegetation metrics, and two fitness-related metrics (corticosterone and body weight) in a free-living common native urban songbird, the American robin (*Turdus migratorius*), at a densely sampled intra-neighborhood scale. Robins can be considered urban exploiters (Reale and Blair, 2005), because their density (Nelson and Nelson, 2001; Suarez-Rubio et al., 2011) and survivorship (Evans et al., 2015; Pharr et al., 2023) can be higher in urban compared to natural areas. In urban areas, however, robins have been shown to be exposed to common urban pollutants, including heavy metals (Roux and Marra, 2007; Zahor et al., 2024).

For greenness and pollution data, we leverage the ongoing large-scale experimental research effort of The Green Heart Louisville Project (GH, greenheartlouisville.com). GH is a one-of-its-kind experimental implementation of urban greenery to test the hypothesis that increasing neighborhood greenness diminishes the risk of cardiovascular disease in humans by decreasing levels of air pollution (Bhatnagar et al., 2023). GH monitors detailed measures of air quality and greenness in a 12 km^2^ study area in Louisville, KY, in tandem with a longitudinal clinical study assessing cardiovascular function and risk factors in area residents. To our knowledge, ours is the first attempt to assess the extent to which various greenness types, air pollutants, and their interaction predicts avian health and physiology.

## Materials and methods

### Study area

We followed all applicable institutional and national guidelines for the care and use of animals. This study was approved the University of Louisville IACUC (protocol number 20844) and was permitted under federal Bird Banding Permit 24288 and Kentucky Dept. Of Fish and Wildlife Resources permit SC2511071. We studied free living American robins (*T. migratorius*) across a contiguous 10 km^2^ residential neighborhood in Louisville, KY (lat: 38.190,266; long: -85.778,094) which encompasses the area of the GH project (Supplementary Figure S1). Our study area is intersected by a major six-lane highway and has high surface road coverage, resulting in a high traffic volume that increases the levels of air and noise pollution. The area has a high human population density (ca. 2,700/km^2^). In addition to single home residences with traditional suburban front and back yards, the area includes several city parks, schools, and local businesses.

### Bird capture and sampling

We caught free-living adult robins (n = 66, 37 males, 29 females) from April 27 to 30 June 2022. We captured the birds during their morning foraging (between 6–9 a.m.), placing standard (60 mm mesh size, 12 or 18 m in length) mist nets in lawns in parks, road medians, school grounds, and private residences across the study area. Most birds (52 out of 66) were caught passively without decoys or playback, with six birds being caught at their nests and eight being caught in response to nestling and adult alarm calls. Permission was obtained from Louisville parks officials, private residence owners, or school officials before bird capture. Upon capture, we immediately collected a blood sample (75 µL) from the brachial vein. We used a 26 G needle to puncture the vein and collected blood in heparinized microcapillary tubes. Blood samples were collected between 72 and 180 s after the bird fell into the mist net. Blood samples were placed on ice and transported to the lab, where they were centrifuged for 10 min to separate red blood cells from plasma. Plasma was then aspirated and frozen at −20°C. After blood collection, we measured the weight of each bird using a 100 g spring scale, which was used as an estimate of the body condition (Green, 2001). Each bird was banded with one USGS aluminum and three plastic color leg bands. Birds were released at the capture location.

### Corticosterone assays

We analyzed corticosterone concentration in the plasma using previously validated enzyme-linked immunosorbent assay for this species (Abolins-Abols and Hauber, 2020). Briefly, we suspended 10 µL plasma in 200 double-distilled water and mixed it with 1 mL diethyl ether (Fisher Scientific, E138-1). The suspension was vortexed, after which ether and aqueous phases were allowed to separate. The aqueous phase was then flash-frozen, and the ether phase decanted. The extraction procedure was repeated three times, following which ether was evaporated under a gentle stream of nitrogen. We then used a commercial corticosterone ELISA kit (Cayman Chemical, 501320) to analyze corticosterone concentration in the extract using manufacturer’s instructions. The plasma extract was suspended in 600 µL assay buffer and vortexed for 1 min before storing it at 4°C overnight. We ran the extracted samples in triplicate, using a pooled robin plasma extract as a within- and across-plate control. We measured plate absorbance at 405 nm. Data were analyzed using a eight-point logistic curve using the analysis spreadsheet provided by Cayman Chemical. Most samples fell within 20%–80% B/Bo range. The among-plate coefficient of variation was 5.79%, whereas the within-plate coefficient of variation was 6.18%. We averaged the data from the three replicates and standardized sample concentrations across plates using the mean concentration of the control samples.

### Air pollution and greenness variables

We measured NO_2_ across the study area with a 60-site passive sampling network (Prathibha et al., 2023). We measured ultrafine particulate matter (UFP) number *via* mobile sampling across the study area roadway network across seasons, days of week, and times of day. For both UFP and NO_2_, we used a land use regression modeling approach to estimate respective spatially resolved annual concentration estimate raster surfaces across the study area (Prathibha, 2021).

To assess NDVI, we collected raster-based multispectral data at 3 m^2^ spatial resolution from Planet Labs DOVE satellite imagery for summer (May 1 – September 30) 2022. Prior to our collection, the raw imagery data was downscaled by Planet Labs from 3.7 to 4.1 m^2^ resolution to a consistent 3 m^2^ resolution. We excluded data from time points with >10% cloud cover, yielding 40 viable time points with a mean time between collection of 3.9 days. We calculated NDVI raster layers for each time point and then created a raster layer of the temporal mean NDVI value of all time points, for all pixels, to compile our final summer NDVI measurement. For leaf area index (LAI), we commissioned a fixed wing LiDAR data collection of the study area in the summer of 2019 and calculated LAI based on the Beer-Lambert law, described elsewhere (Yeager et al., 2023). The resulting LAI values represented estimated total leaf area, quantified as a 2-dimensional value of the 3-dimensional sum, per square meter. To identify areas of grass, we also collected seven in resolution multispectral data during the summer 2019 fixed-wing data collection (Yeager et al., 2024). From seven in imagery, we calculated NDVI, removed building and canopy footprints, and identified and extracted areas of grass based on distinct NDVI cut points that differentiates areas of grass from impervious surfaces and other vegetation. We then created a dichotomous raster surface, coded as 0 and 100 to represent grass cover at seven in resolution, to classify pixels of grass cover.

For spatial aggregation of all exposure data (NO_2_, UFP, NDVI, LAI, grass), we used the Focal Statistics tool in ArcGIS Pro software to create secondary raster surfaces with pixel values calculated as the mean value of the original raster surface within each given radius. The resulting secondary raster surfaces represent a mean of continuous measurement values within a given radius except for grass represented as percent coverage within respective radii. From secondary mean radii raster surfaces, we extracted radii values for each exposure at the sampling location.

### Statistical analyses

All analyses were conducted in R, version 4.4.1 (R Core Team, 2024). Corticosterone values were right-skewed and were therefore natural log-transformed. Log-transformed corticosterone values showed a positive relationship with the length of time elapsed between capture and completion of the blood sample (Linear Model (LM), df = 65, slope = 0.01, multiple *r* ^2^ = 0.12, p = <0.01), indicating that some individuals might have started to secrete corticosterone in response to capture stress within 3 min after capture. We therefore calculated residuals from a regression between corticosterone values and the time it took to complete the collection of the blood sample and used these residuals as the response variable in analyses of corticosterone levels. Corticosterone was not related to bird capture type (ANOVA, df = 61, F = 8.60, p = 0.52) or body weight (LM, df = 65, slope = −0.01, multiple *r* ^2^ = <0.01, p = 0.54).

To ask what environmental factors best explained variation in the corticosterone residuals and body condition, we used AIC-based model selection approach followed by model averaging (Burnham et al., 2011; Symonds and Moussalli, 2011). AIC-based model selection allows for non-biased model generation for correlative datasets in natural systems (Aho et al., 2014). This approach can be especially useful when more than one independent candidate predictors may explain variation in the response variable, but where a model that includes every covariate may not necessarily be the best model. However, AIC-based model selection can produce several similar, competitive models. Model averaging of competing models allows evaluating the strength of evidence supporting a relationship between independent variables included in these competing models and the response variable. To identify the best models, we used the package *MuMIn* (Bartoń, 2018) to calculate the corrected Akaike information criterion (AICc) scores for linear models that contained different combinations of predictors. We then asked if the top models (within two AICc of the best model) predicted the data significantly better than a null model using likelihood ratio tests using package lmtest (Zeileis and Hothorn, 2002). We then used model averaging for the top models with a cumulative Akaike weight of 0.95 (Burnham et al., 2011; Symonds and Moussalli, 2011) to derive weighted averages of parameter and error estimate across multiple competing models.

Predictors for baseline corticosterone residuals and body weight included air pollution metrics (nitrogen dioxide levels, ultrafine particle levels), greenness metrics (normalized difference vegetation index, leaf area index, and grass cover), capture date, and bird sex. Additionally, we included interactions between all combinations of greenness metrics and air pollution metrics, because we reasoned that birds may respond to air pollution differently across a greenness gradient. We also included an interaction between sex and all environmental variables, because females are the only incubating sex and might show different space use than males during the breeding season.

American robin territory size is 0.11–0.21 ha [but can be as large as 0.84 ha (Howell, 1942; Pitts, 1984)], but their home range can encompass a much greater area [1.3–14,417.1 ha (Benson et al., 2012)]. We therefore first analyzed the relationship between the response variables and averaged environmental variables at two different radii from their capture location: 20 m (representing an area of 0.13 ha, consistent with territory size) and 500 m (78.54 ha, consistent with a small home range). Because the entire GH study area covers approximately 1,000 ha (Yeager et al., 2024), an area around the capture location with a larger radius (e.g., 1,000 m, consistent with a large robin home range) would cover most of study area and result in a loss of resolution. It is important to note, however, that we do not know if the capture location for each individual fell within its territory or how representative the capture location was of the average space use for each individual. We treat these two spatial scales as hypothesis-driven starting points in our analyses.

Following initial analyses using environmental values at 20 m and 500 m radii, we calculated standardized slope estimates for each environmental variable across these and intermediate distances (50 m, 100 m, 200 m, 300 m, 400 m) to better understand how the relationship between environmental variables and response variables (corticosterone or body weight) may change with distance. In these models, we considered each environmental variable separately. For corticosterone, we also included date as a covariate. Because model selection identified sex-dependent associations between the response variables and environmental variables, we then recalculated the effect sizes for both sexes separately.

Variance inflation analyses identified that the greenness variables were strongly correlated. In particular, in our dataset grass cover and leaf area index were strongly negatively (*r* = −0.75) correlated at the 20 m radius. At the 500 m radius, leaf area index was strongly positively (*r* = 0.76) correlated with NDVI. However, model selection is not sensitive to multicollinearity (Graham, 2003; Pipoly et al., 2022) because models are considered separately for their fit. Including collinear variables, such as leaf area index and grass cover, in the full model allows comparing the fit of smaller models with alternative collinear variables. Because more than one greenness metric was present in the top models, we report conditional (as opposed to full) model-averaged coefficients and p-values.

## Results

### Summary statistics

Summary statistics for the response and independent variables are reported in Supplementary Table S1. Environmental variables showed stronger variation at the smaller (20 m) radius around the capture location compared to the larger radius (500 m). At 20 m from bird capture locations, the difference between minimum and maximum greenness indices ranged from 7-fold (for NDVI) up to 120-fold (for LAI). The fold difference between minimum and maximum pollutant exposure at this radius ranged from 3.9-fold (for NO2) to 6-fold (for UFPs). At 500 m from bird capture locations, we saw lower variation in environmental variables, especially for pollutants.

### Corticosterone

**FIGURE 1:**
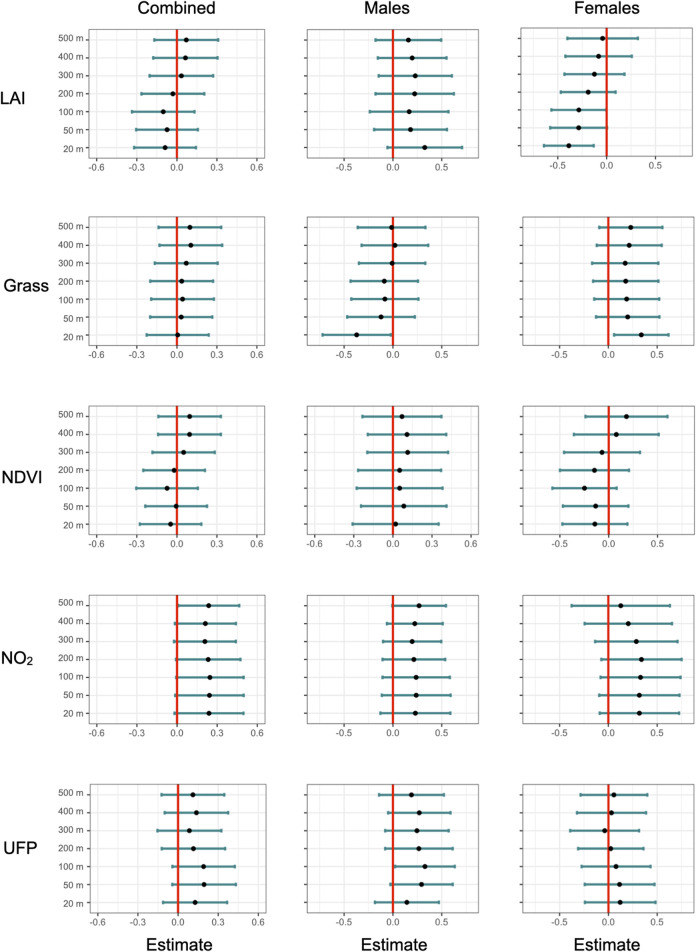
Standardized slope estimates of environmental variables predicting corticosterone at increasing radii from the capture location. Rows represent environmental variables, columns represent wither models with box sexes or each sex separately. Error bars represent the 95% confidence interval (CI) for the slope estimate.

**FIGURE 2:**
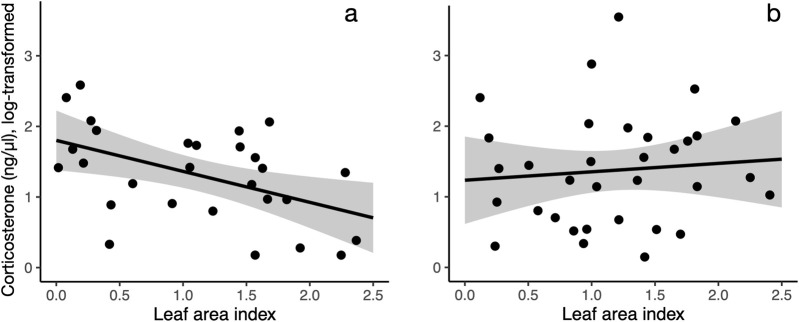
The relationship between corticosterone and leaf area index differs in females **(a)** and males **(b)**. Leaf area index (20 m radius from capture location) is negatively correlated with corticosterone in females **(a)** but not in males **(b)**. Shaded area indicates 95% confidence interval for the line.

Our measurements of plasma corticosterone showed that in the area with a 20 m radius from the capture location, variation in baseline corticosterone was best explained by date and an interaction between sex and greenness variables (LAI or grass). After model selection, seven top models (of out 7,260 total, Supplementary Table S2) were within 2.0 AICc-s of each other, indicating that statistical support for these models was equally strong. These models included either an interaction between LAI and bird sex (increasing LAI was associated with lower corticosterone in females but not in males, Figures 1 and 2) or grass and bird sex (increasing grass area was associated higher corticosterone in females but lower corticosterone in males). Since LAI was strongly negatively associated with grass at the 20 m radius, the bird sex-dependent relationships between corticosterone and the two different greenness metrics are likely not independent but represent two alternative hypotheses. In the model with the lowest AICc, the interaction between LAI and sex was significant, as was the effect of date and the main effect of LAI (Table 1). The interaction between LAI and sex as well as and grass and sex remained significant after model averaging of the models with combined AICc weight of 0.95 (Supplementary Table S3).

**TABLE 1:** Top model predicting corticosterone using environmental variables at 20 m radius from the capture location.

Factor	Estimate (95% CI)	SE	*t*	*p*
Intercept	0.23 (−0.09, 0.55)	0.16	1.42	0.16
DATE	−0.37 (−0.63, −0.12)	0.13	−2.92	<0.01
NO_2_	0.21 (−0.05, 0.46)	0.13	1.63	0.11
LAI	−0.35 (−0.65, −0.05)	0.15	−2.34	0.02
SEX (male)	−0.42 (−0.85, 0.02)	0.22	−1.93	0.06
LAI ✕ SEX (male)	0.66 (0.22, 1.10)	0.22	3.00	<0.01

*Note*. CI, confidence interval; SE, standard error; DATE, day since January 1; SEX, male or female; LAI, leaf area index; NO_2_, nitrogen dioxide concentration.

At 500 m radius from the capture location, variation in baseline corticosterone was best explained by date and NO_2_ concentration. After model selection, five top models (out of 7,260 total, Supplementary Table S4) were within 2.0 AICc-s of each other. These models showed that higher levels of plasma corticosterone were positively associated with ambient NO_2_ concentrations (Figure 1). NO_2_ was present in all five top models, and the relationship between corticosterone and NO_2_ was significant in the model with the lowest AICc (Table 2). However, the relationship between NO_2_ and corticosterone was not significant after model averaging of the models with combined AICc weight of 0.95 (Supplementary Table S5).

**TABLE 2:** Top model predicting corticosterone using environmental variables at 500 m radius from the capture location.

Factor	Estimate (95% confidence interval)	SE	*t*	*p*
Intercept	0.00 (−0.22, 0.22)	0.11	0.00	1.00
DATE	−0.35 (−0.58, −0.13)	0.11	−3.15	<0.01
NO_2_	0.27 (0.05, 0.50)	0.11	2.42	0.02

*Note*. CI, confidence interval; SE, standard error; DATE, day since January 1; NO_2_, nitrogen dioxide concentration.

### Body condition

**FIGURE 3:**
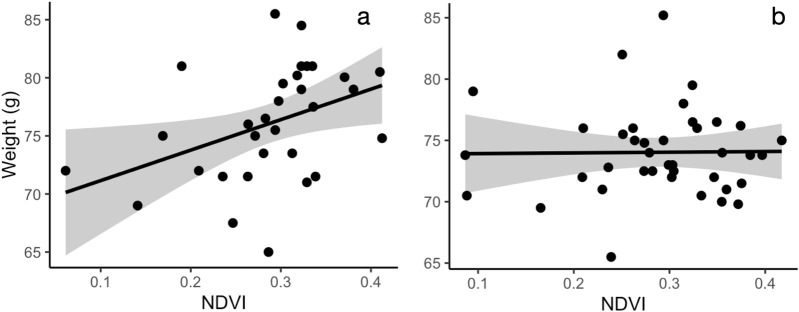
The relationship between body weight and NDVI in females **(a)** and males **(b)**. Leaf area index (20 m radius from capture location) is positively correlated with weight in females **(a)** but not in males **(b)**. Shaded area indicates 95% confidence interval for the line.

In the area with a 20 m radius from the capture location, variation in body condition, assessed as body weight, was best explained by bird sex and its interaction with NDVI. After model selection, four top models (out of 7,260 total) were within 2.0 AICc-s of each other. All but one of the top models showed that increasing NDVI was associated with higher body weight in females but not in males (Figure 3; Supplementary Table S6). In the model with the lowest AICc (Table 3), the main effect of NDVI showed a significant positive association with body weight, but this relationship was modified by the significant negative interaction term between NDVI and male sex. The interaction between NDVI and sex remained marginally significant after model averaging of the models with the combined AICc weight of 0.95 (Supplementary Table S7). At 500 m radius from the capture location, none of top models for body weight that included environmental data were significantly better than the null model (Supplementary Figure S2; Supplementary Table S8).

**TABLE 3:** Top model predicting body weight using environmental variables at 20 m radius from the capture location.

Factor	Estimate (95% CI)	SE	*t*	*p*
Intercept	0.26 (−0.08, 0.60)	0.17	1.54	0.13
SEX	−0.47 (−0.92, −0.02)	0.23	−2.07	0.04
NDVI	0.47 (0.12, 0.83)	0.18	2.65	0.01
NDVI ✕ SEX	−0.46 (−0.92, 0.00)	0.23	−2.00	0.05

Note. CI = confidence interval; SE = standard error; SEX = male or female; NDVI = normalized difference vegetation index.

## Discussion

In this study we show that in a native urban songbird at an intra-neighborhood scale, baseline corticosterone is negatively associated with canopy volume and positively associated with pollution exposure, while body weight has positively associated with overall greenness. However, the associations between environmental and physiological metrics depended on bird sex and the scale at which the environmental metrics were calculated.

We found the strongest associations between vegetation, body weight, and corticosterone at smaller spatial scales. This is concordant with a previous study in this species that show that robin distribution is best predicted by vegetation characteristics at a small spatial scale (Pennington and Blair, 2011). Specifically, we found that female, but not male, birds had lower baseline corticosterone in areas with high leaf area index. However, this relationship was significant only near the capture location (area with a 20 m radius around the capture location, roughly representing robin territory size, Figure 1), but was not apparent when considering the average leaf area index in areas with larger radii. Similarly, body weight was positively associated with NDVI in female, not male birds (Figure 3), and this relationship was only apparent when considering greenness near the capture location (Supplementary Figure S2).

The sex difference in the relationship between leaf area and corticosterone, and overall greenness and body condition, may be related to potentially different space use of robins in the summer. Breeding robin females spend up to 14 days incubating eggs (Vanderhoff et al., 2016) and therefore have prolonged interactions with the environment immediately around the nest. This is in contrast to male robins, which do not incubate and can move around more freely. To our knowledge, it is not known whether robin females and males use space differently when females are not incubating. However, recent studies highlight the fact that factors associated with urban development may affect male and female birds differently (Lane et al., 2023).

There are multiple possible explanations for the relationship between corticosterone levels, urban vegetation and sex. In our dataset, leaf area index was negatively correlated with grass area. Models with interactions between either bird sex and leaf area index or between sex and grass cover were within 2 AICc’s, indicating that, statistically, these models had very similar explanatory power. Higher leaf area index has been shown to be a strong driver of reduced temperatures (Hardin and Jensen, 2007; Bakhshoodeh et al., 2022), which may reduce the metabolic load of thermoregulation during warm summer days. However, data on heart rate, an indicator of metabolic rate (Steiger et al., 2009), from the same study site obtained with a different bird cohort (unpublished), suggest that high ambient temperatures within the range encountered in the neighborhood are not associated with increased metabolic rate in robins. Alternatively, higher leaf area may shield birds from potential stressors, such as humans, racoons, and domestic cats, resulting in a reduced corticosterone (Scheuerlein et al., 2001). It is also possible that the relationship between vegetation and corticosterone levels may be related to food abundance: in general, lower food availability is associated with elevated corticosterone in birds (Mench, 1991; Kitaysky et al., 2001; Lynn et al., 2003; Schoech et al., 2004). Because foliage is one of the main substrates for insect foraging by American robins (Paszkowski, 1982), increasing leaf area may be linked to higher prey abundance and, as a consequence, lower corticosterone. On the other hand, the positive association of grass area with corticosterone levels is less intuitive. One possibility is that the relationship between corticosterone and vegetation in female birds may be linked to the habitat preference of haemosporidian parasite vectors (biting midge and mosquitoes). However, studies show variable relationship between haemosporidian infection and corticosterone (Garvin and Schoech, 2006; Schoenle et al., 2017). While it is not possible to determine if leaf area or grass is driving the relationship between sex, vegetation, and corticosterone in this dataset, it is important to note that it was not the total greenness (assessed using NDVI) driving this relationship, but that corticosterone was associated with a specific type of urban vegetation. This suggests that studies in urban avian ecology should not treat vegetation as a homogenous variable but parse the effects of different vegetation types on avian physiology and health.

The positive association between the overall greenness, measured as NDVI, and female body condition, could be driven by increased food abundance in greener areas. Alternatively, heavier females might outcompete lighter females for greener territories. More generally, the alternative interpretations for these relationships highlight the inherent limitation of the correlative approach used in this study. Indeed, it is possible that these relationships are driven by another, unmeasured variable. However, we view these relationships are useful starting points for future experimental or pre-post studies.

Corticosterone levels were positively associated with elevated levels of ambient nitrogen dioxide, which is a proxy for traffic-generated pollutants. The relationship between nitrogen dioxide and corticosterone was largely the same when considering the average NO_2_ concentration across distances from the capture location, but model selection suggested that NO_2_ was an important predictor of corticosterone only when averaged across an area representative of a robin home range (area with a 500 m radius around the capture location). In these models, NO_2_ concentration was included in the top model, where it was significantly positively associated with corticosterone levels, irrespective of sex. However, the *r* ^2^ of this model was only 0.20. Furthermore, model averaging showed that support for this relationship is weak, because the positive NO_2_-corticosterone association was not significant after model averaging of the top models with Akaike weight of 0.95. NO_2_ is a strong oxidizing agent leading to the formation of ozone through interaction with sunlight (Sicolo et al., 2010; Sanderfoot and Holloway, 2017). Ozone damages lung epithelium and causes capillary inflammation in birds (Rombout et al., 1991). Experimental exposure to a mix of air pollutants, including NO_2_, results in elevated corticosterone and oxidative stress markers in kestrels (Cruz-Martinez et al., 2015). While the evidence for the relationship between NO_2_ and corticosterone in our field study is limited, it nevertheless supports the results from experimental lab studies on the effect of NO_2_ on corticosterone. It is also possible that the relationship between NO_2_ and corticosterone is mediated by another factor related to vehicular traffic, such as traffic noise, which has been linked to changes in the hypothalamic-pituitary-adrenal axis activity in wild songbirds (Injaian et al., 2018).

Direct comparisons between avian and human health markers are not straight forward due to the differences between birds and humans in space use, diet, and physiology. However, it is important to note that in the same Green Heart Louisville (GH) study area leaf area index was negatively associated with blood pressure (Yeager et al., 2024). The overall greenness, measured as NDVI, in the GH area was associated with lower inflammation (Sears et al., 2024). NDVI was also associated with reduced sympathetic activation and inflammation city-wide in Louisville (Yeager et al., 2018). Although these associations are different from what we report here, they suggest that in both birds and humans, urban vegetation may be associated with reduced stress-response markers.

It is important to note that our findings are limited to one species in one location during the breeding season and that this study has limited sample size. A potential additional limitation of the interpretation of our results is that the environment at or around the capture area might not accurately represent the average environment the captured birds experienced. As outlined above, while robin territory size is 0.11–0.21 ha [but can be as large as 0.84 ha (Howell, 1942; Pitts, 1984)], their home ranges, which are rarely spherical, can encompass a much greater area (1.3–14,417.1 ha; Benson et al., 2012). These limitations can be addressed in future by conducting longer-term studies on incubating females, nestlings, or species with smaller home ranges.

In summary, we demonstrate that, in a native urban songbird at an intra-neighborhood level, greenness shows sex-dependent associations with body weight and corticosterone, and air pollution is positively associated with corticosterone. This suggests that avian stress physiology and fitness may differ not only across a broad urban-rural gradient, but at a much finer scale within and between neighborhoods. This, in turn, suggests that conservation efforts of urban wildlife should be directed to modifying urban green architecture and pollution dispersion.

## Data Availability

The raw data supporting the conclusions of this article will be made available by the authors, without undue reservation.
