# Supplementary material for: Greenness and pollution exposure predict corticosterone concentration in an urban songbird
Source: Front Physiol. 2025 Jun 25;16:1603811. doi: 10.3389/fphys.2025.1603811 (PMC12238760; doi:10.3389/fphys.2025.1603811)
Supplement: Supplementary file 1 [file DataSheet1.docx]

**Supplementary material for:**

Greenness and pollution exposure predict corticosterone concentration in an urban songbird.

**Authors:**

Mikus Abolins-Abols. Ray Yeager, Jay Turner, Ted Smith, Aruni Bhatnagar

Supplementary Figure 1: Capture locations of adult American robins (*Turdus migratorius*) in this study. The black like delineates the Green Heart environmental health study area.


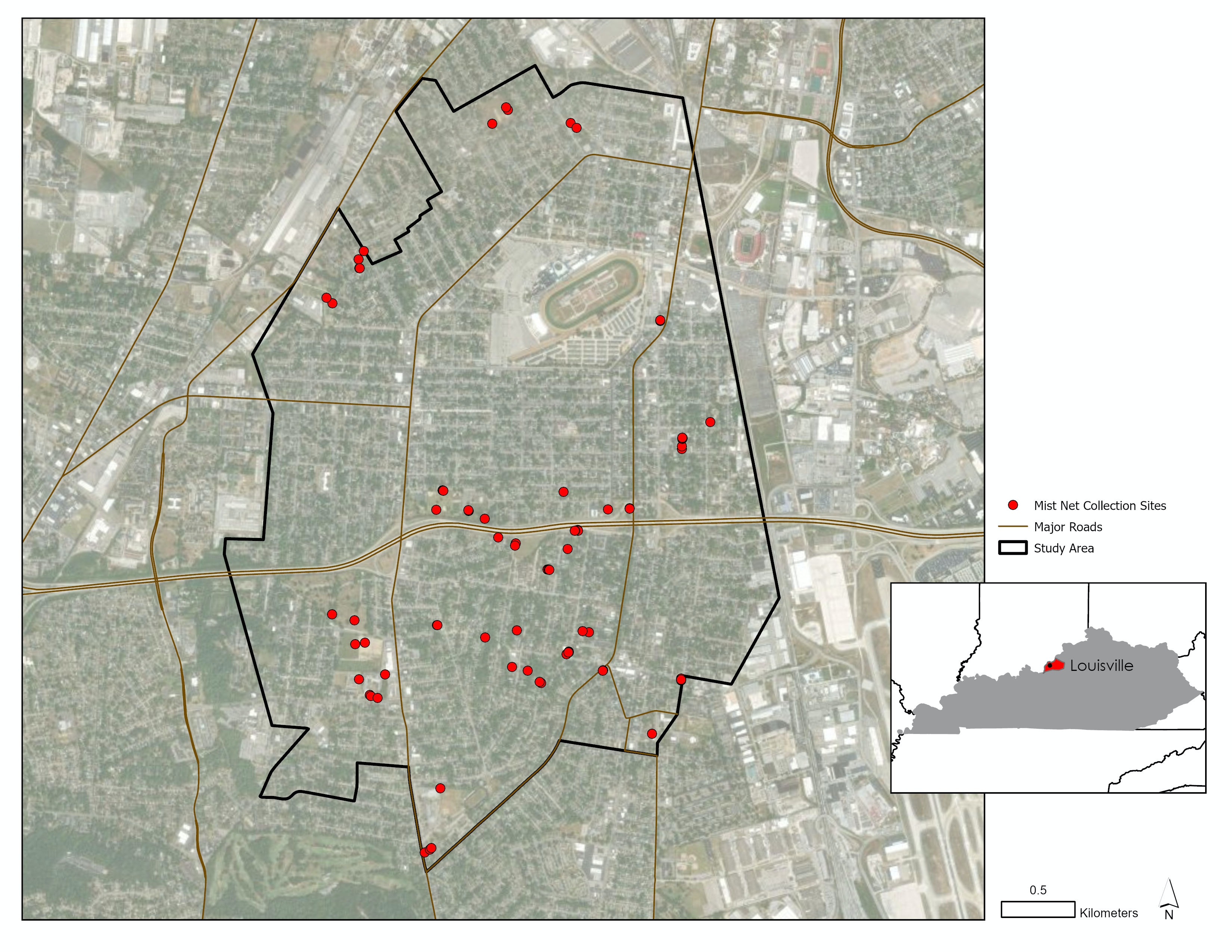


**Supplementary Table 1**: *Summary statistics for dependent and independent variables in the study*

| Variable | Units | n | Median | Mean | Variance | St. Dev. | Min. value | 25th quartile | 75th quartile | Max. value |
| --- | --- | --- | --- | --- | --- | --- | --- | --- | --- | --- |
| Cort | ng/ul | 66 | 3.50 | 4.80 | 25.11 | 5.01 | 0.47 | 1.78 | 5.94 | 34.67 |
| SMI | NA | 70 | 74.83 | 74.99 | 27.31 | 5.23 | 64.19 | 71.80 | 77.64 | 87.75 |
| LA at 20 m | NA | 70 | 1.21 | 1.15 | 0.44 | 0.66 | 0.02 | 0.58 | 1.66 | 2.41 |
| LA at 500 m | NA | 70 | 0.95 | 0.96 | 0.02 | 0.15 | 0.52 | 0.85 | 1.04 | 1.24 |
| Grass at 20 m | % cover | 70 | 0.35 | 0.38 | 0.05 | 0.22 | 0.09 | 0.21 | 0.54 | 0.90 |
| Grass at 500 m | % cover | 70 | 0.25 | 0.26 | 0.00 | 0.04 | 0.20 | 0.22 | 0.29 | 0.35 |
| NDVI at 20 m | NA | 70 | 0.30 | 0.29 | 0.01 | 0.08 | 0.06 | 0.25 | 0.34 | 0.42 |
| NDVI at 500 m | NA | 70 | 0.22 | 0.22 | 0.00 | 0.04 | 0.09 | 0.20 | 0.23 | 0.29 |
| NO_2_ at 20 m | ppb | 70 | 13.07 | 15.08 | 31.03 | 5.57 | 7.02 | 11.90 | 19.86 | 27.46 |
| NO_2_ at 500 m | ppb | 70 | 14.83 | 14.28 | 2.33 | 1.53 | 9.58 | 14.57 | 15.11 | 15.42 |
| UFP at 20 m | #/cm3 | 70 | 8271.14 | 9054.10 | 13492270.79 | 3673.18 | 3930.29 | 6805.33 | 10693.40 | 24607.46 |
| UFP at 500 m | #/cm3 | 70 | 9149.16 | 8928.55 | 464925.75 | 681.85 | 7663.58 | 8370.10 | 9450.86 | 9993.27 |

**Supplementary Table 2**: *Best models predicting corticosterone using environmental variables at 20 m radius from the capture location after model selection.*

| *Model* | k | AICc | ΔAICc | w_i_ | *p* | *r^2^* |
| --- | --- | --- | --- | --- | --- | --- |
| DATE + SEX + LAI + NO_2_ + LAI ✕ SEX | 5 | 176.5 | 0 | 0.05 | <0.01 | 0.32 |
| DATE + SEX + LAI + LAI ✕ SEX | 4 | 176.9 | 0.37 | 0.04 | <0.01 | 0.29 |
| DATE + SEX + LAI + NO_2_ + LAI ✕ SEX + NO_2_ ✕ SEX | 6 | 177.2 | 0.74 | 0.03 | <0.01 | 0.34 |
| DATE + SEX + GRASS + GRASS ✕ SEX | 4 | 177.2 | 0.75 | 0.03 | <0.01 | 0.29 |
| DATE + SEX + GRASS + NO_2_ + GRASS ✕ SEX | 5 | 177.8 | 1.27 | 0.02 | <0.01 | 0.31 |
| DATE + SEX + LAI + GRASS + LAI ✕ SEX | 5 | 178.5 | 1.98 | 0.02 | <0.01 | 0.30 |

*Note*. k = number of fixed terms in the model; AICc = Akaike Information Criterion, ΔAICc = difference in AICc between the focal model and the best model; w_i_ = Akaike weights; p = probability that the model explains more variation than the null model; r^2^ = multiple r^2^; DATE = day since Jan 1; SEX = male or female; LAI = leaf area index; NO_2_ = nitrogen dioxide concentration; GRASS = grass area.

**Supplementary Table 3**: *Averaged parameter estimates predicting corticosterone using environmental variables at 20 m radius from the capture location using models with cumulative AIC weight of 0.95.*

| Factor | Estimate | CI of estimate | Adj. SE | z | p |
| --- | --- | --- | --- | --- | --- |
| (Intercept) | 0.21 | [-0.13, 0.55] | 0.17 | 1.20 | 0.23 |
| **DATE** | **-0.41** | **[-0.68, -0.13]** | **0.14** | **2.93** | **<0.01** |
| LAI | -0.39 | [-0.89, 0.11] | 0.25 | 1.54 | 0.12 |
| NO_2_ | 0.14 | [-0.22, 0.50] | 0.18 | 0.76 | 0.45 |
| SEX (male) | -0.41 | [-0.86, 0.04] | 0.23 | 1.80 | 0.07 |
| **LAI** ✕ **SEX (male)** | **0.67** | **[0.14, 1.20]** | **0.27** | **2.47** | **0.01** |
| NO_2_ ✕ SEX (male) | 0.26 | [-0.23, 0.74] | 0.25 | 1.04 | 0.30 |
| GRASS | 0.06 | [-0.54, 0.66] | 0.31 | 0.20 | 0.84 |
| **GRASS** ✕ **SEX (male)** | **-0.58** | **[-1.15, 0.00]** | **0.29** | **1.97** | **0.05** |
| UFP | 0.12 | [-0.18, 0.42] | 0.15 | 0.79 | 0.43 |
| LAI ✕ UFP | -0.27 | [-0.72, 0.17] | 0.23 | 1.20 | 0.23 |
| LAI ✕ NO_2_ | -0.08 | [-0.35, 0.19] | 0.14 | 0.60 | 0.55 |
| NDVI | 0.10 | [-0.30, 0.50] | 0.20 | 0.51 | 0.61 |
| GRASS ✕ UFP | 0.20 | [-0.20, 0.60] | 0.20 | 0.99 | 0.32 |
| GRASS ✕ NO_2_ | -0.03 | [-0.35, 0.30] | 0.17 | 0.17 | 0.87 |
| NDVI ✕ SEX (male) | 0.01 | [-0.64, 0.65] | 0.33 | 0.02 | 0.98 |
| SEX ✕ UFP | 0.06 | [-0.40, 0.51] | 0.23 | 0.25 | 0.80 |
| NDVI ✕ NO_2_ | -0.05 | [-0.32, 0.21] | 0.13 | 0.39 | 0.69 |
| NDVI ✕ UFP | 0.02 | [-0.49, 0.53] | 0.26 | 0.06 | 0.95 |

*Note*. CI = confidence interval; SE = standard error; DATE = day since Jan 1; NO_2_ = nitrogen dioxide concentration; UFP = ultrafine particulate concentration; SEX = male or female; LAI = leaf area index; NDVI = normalized difference vegetation index; GRASS = grass area.

**Supplementary Table 4**: *Best models predicting corticosterone using environmental variables at 500 m radius from the capture location after model selection.*

| *Model* | k | AICc | ΔAICc | w_i_ | *p* | *r^2^* |
| --- | --- | --- | --- | --- | --- | --- |
| DATE + NO_2_ | 2 | 179.8 | 0 | 0.06 | <0.01 | 0.20 |
| DATE + NO_2_ + SEX (male) | 3 | 180.1 | 0.27 | 0.05 | <0.01 | 0.23 |
| DATE + NO_2_ + GRASS | 3 | 180.8 | 1 | 0.04 | <0.01 | 0.22 |
| DATE + NO_2_ + GRASS + SEX (male) | 4 | 181.7 | 1.85 | 0.02 | <0.01 | 0.24 |
| DATE + NO_2_ + NDVI | 3 | 181.8 | 1.96 | 0.02 | <0.01 | 0.21 |

*Note*. k = number of fixed terms in the model; AICc = Akaike Information Criterion, ΔAICc = difference in AICc between the focal model and the best model; w_i_ = Akaike weights; p = probability that the model explains more variation than the null model; r^2^ = multiple r^2^; DATE = day since Jan 1; SEX = male or female; NO_2_ = nitrogen dioxide concentration; GRASS = grass area; NDVI = normalized difference vegetation index.

**Supplementary Table 5**: *Averaged parameter estimates predicting corticosterone using environmental variables at 500 m radius from the capture location using models with cumulative AIC weight of 0.95.*

| Factor | Estimate | CI of estimate | Adj. SE | z | p |
| --- | --- | --- | --- | --- | --- |
| Intercept | 0.11 | [-0.25, 0.48] | 0.18 | 0.62 | 0.54 |
| **DATE** | **-0.37** | **[-0.61, -0.13]** | **0.12** | **3.03** | **<0.01** |
| NO_2_ | 0.24 | [-0.11, 0.59] | 0.18 | 1.34 | 0.18 |
| SEX (male) | -0.33 | [-0.82, 0.15] | 0.25 | 1.34 | 0.18 |
| GRASS | 0.19 | [-0.27, 0.65] | 0.23 | 0.79 | 0.43 |
| NDVI | 0.03 | [-0.63, 0.70] | 0.34 | 0.10 | 0.92 |
| UFP | 0.03 | [-0.31, 0.37] | 0.17 | 0.19 | 0.85 |
| NO_2_ ✕ SEX | 0.19 | [-0.44, 0.82] | 0.32 | 0.59 | 0.55 |
| LAI | 0.07 | [-0.60, 0.73] | 0.34 | 0.20 | 0.84 |
| GRASS ✕ NO_2_ | -0.14 | [-0.58, 0.30] | 0.22 | 0.62 | 0.53 |
| GRASS ✕ SEX (male) | -0.25 | [-0.75, 0.26] | 0.26 | 0.96 | 0.34 |
| NDVI ✕ SEX (male) | -0.23 | [-0.85, 0.40] | 0.32 | 0.72 | 0.47 |
| NDVI ✕ NO_2_ | -0.02 | [-0.47, 0.43] | 0.23 | 0.09 | 0.93 |
| LAI ✕ NO_2_ | 0.09 | [-0.45, 0.62] | 0.27 | 0.32 | 0.75 |
| UFP ✕ SEX (male) | -0.16 | [-0.70, 0.38] | 0.27 | 0.58 | 0.56 |
| LAI ✕ SEX (male) | 0.09 | [-0.54, 0.72] | 0.32 | 0.28 | 0.78 |
| GRASS ✕ UFP | -0.09 | [-0.42, 0.23] | 0.17 | 0.56 | 0.57 |
| LAI ✕ UFP | 0.15 | [-0.16, 0.47] | 0.16 | 0.95 | 0.34 |
| NDVI ✕ UFP | 0.10 | [-0.19, 0.39] | 0.15 | 0.66 | 0.51 |

*Note*. CI = confidence interval; SE = standard error; DATE = day since Jan 1; NO_2_ = nitrogen dioxide concentration; UFP = ultrafine particulate concentration; SEX = male or female; LAI = leaf area index; NDVI = normalized difference vegetation index; GRASS = grass area.

**Supplementary Table 6**: *Best models predicting body weight using environmental variables at 20 m radius from the capture location after model selection.*

| *Model* | k | AICc | ΔAICc | w_i_ | *p* | *r^2^* |
| --- | --- | --- | --- | --- | --- | --- |
| NDVI + SEX + NDVI ✕ SEX | 3.00 | 197.50 | 0.00 | 0.44 | 0.01 | 0.15 |
| NDVI + SEX + NDVI ✕ SEX + GRASS | 4.00 | 198.80 | 1.39 | 0.22 | 0.02 | 0.16 |
| NDVI + SEX | 2.00 | 199.20 | 1.78 | 0.18 | 0.03 | 0.10 |
| NDVI + SEX + NDVI ✕ SEX + LAI | 4.00 | 199.40 | 1.90 | 0.17 | 0.02 | 0.15 |

*Note*. k = number of fixed terms in the model; AICc = Akaike Information Criterion, ΔAICc = difference in AICc between the focal model and the best model; w_i_ = Akaike weights; p = probability that the model explains more variation than the null model; r^2^ = multiple r^2^; NDVI = normalized difference vegetation index; SEX = male or female; GRASS = grass area; LAI = leaf area index.

**Supplementary Table 7**: *Averaged parameter estimates predicting body weight using environmental variables at 500 m radius from the capture location using models with cumulative AIC weight of 0.95.*

| Factor | Estimate | CI of estimate | Adj. SE | z | p |
| --- | --- | --- | --- | --- | --- |
| (Intercept) | 0.25 | [-0.13, 0.62] | 0.19 | 1.28 | 0.20 |
| NDVI | 0.35 | [-0.08, 0.78] | 0.22 | 1.61 | 0.11 |
| SEX (male) | -0.49 | [-0.95, -0.02] | 0.24 | 2.03 | 0.04 |
| NDVI ✕ SEX (male) | -0.45 | [-0.93, 0.03] | 0.24 | 1.84 | 0.07 |
| GRASS | -0.12 | [-0.49, 0.26] | 0.19 | 0.61 | 0.54 |
| LA | 0.11 | [-0.33, 0.56] | 0.23 | 0.49 | 0.62 |
| Date | -0.08 | [-0.33, 0.18] | 0.13 | 0.60 | 0.55 |
| UFP | -0.02 | [-0.29, 0.25] | 0.14 | 0.15 | 0.88 |
| NO_2_ | 0.01 | [-0.28, 0.29] | 0.15 | 0.04 | 0.97 |
| LAI ✕ SEX (male) | -0.19 | [-0.79, 0.41] | 0.30 | 0.62 | 0.53 |
| GRASS ✕ SEX (male) | -0.05 | [-0.63, 0.53] | 0.29 | 0.17 | 0.87 |
| NDVI ✕ NO_2_ | 0.07 | [-0.19, 0.32] | 0.13 | 0.52 | 0.61 |
| NDVI ✕ UFP | 0.12 | [-0.33, 0.56] | 0.22 | 0.51 | 0.61 |
| UFP ✕ SEX (male) | 0.09 | [-0.39, 0.57] | 0.24 | 0.37 | 0.71 |
| NO_2_ ✕ SEX (male) | 0.07 | [-0.42, 0.56] | 0.25 | 0.28 | 0.78 |
| LAI ✕ NO_2_ | 0.11 | [-0.15, 0.37] | 0.13 | 0.82 | 0.41 |
| GRASS ✕ NO_2_ | -0.05 | [-0.33, 0.24] | 0.14 | 0.31 | 0.76 |
| GRASS ✕ UFP | 0.03 | [-0.32, 0.38] | 0.18 | 0.17 | 0.87 |
| LAI ✕ UFP | 0.1 | [-0.32, 0.51] | 0.21 | 0.45 | 0.65 |

*Note*. CI = confidence interval; SE = standard error; DATE = day since Jan 1; NO_2_ = nitrogen dioxide concentration; UFP = ultrafine particulate concentration; SEX = male or female; LAI = leaf area index; NDVI = normalized difference vegetation index; GRASS = grass area.

**Supplementary Table 8**: *Best models predicting body weight using environmental variables at 500 m radius from the capture location after model selection.*

| *Model* | k | AICc | ΔAICc | w_i_ | *p* | *r^2^* |
| --- | --- | --- | --- | --- | --- | --- |
| SEX | 1 | 200 | 0 | 0.33 | 0.04 | 0.06 |
| SEX + UFP | 2 | 201.5 | 1.55 | 0.15 | 0.09 | 0.07 |
| SEX + GRASS | 2 | 201.6 | 1.68 | 0.14 | 0.10 | 0.06 |
| Null model | 0 | 201.8 | 1.86 | 0.13 | NA | NA |
| SEX + LAI | 2 | 201.9 | 1.89 | 0.13 | 0.11 | 0.06 |
| SEX + DATE | 2 | 201.9 | 1.93 | 0.13 | 0.11 | 0.06 |

*Note*. k = number of fixed terms in the model; AICc = Akaike Information Criterion, ΔAICc = difference in AICc between the focal model and the best model; w_i_ = Akaike weights; p = probability that the model explains more variation than the null model; r^2^ = multiple r^2^; NDVI = normalized difference vegetation index; SEX = male or female; GRASS = grass area; LAI = leaf area index.

**Supplementary Figure 2:** *Standardized slope estimates of environmental variables predicting body weight at increasing radii from the capture location. Rows represent environmental variables, columns represent wither models with box sexes or each sex separately. Error bars represent the 95% confidence interval (CI) for the slope estimate.*

**
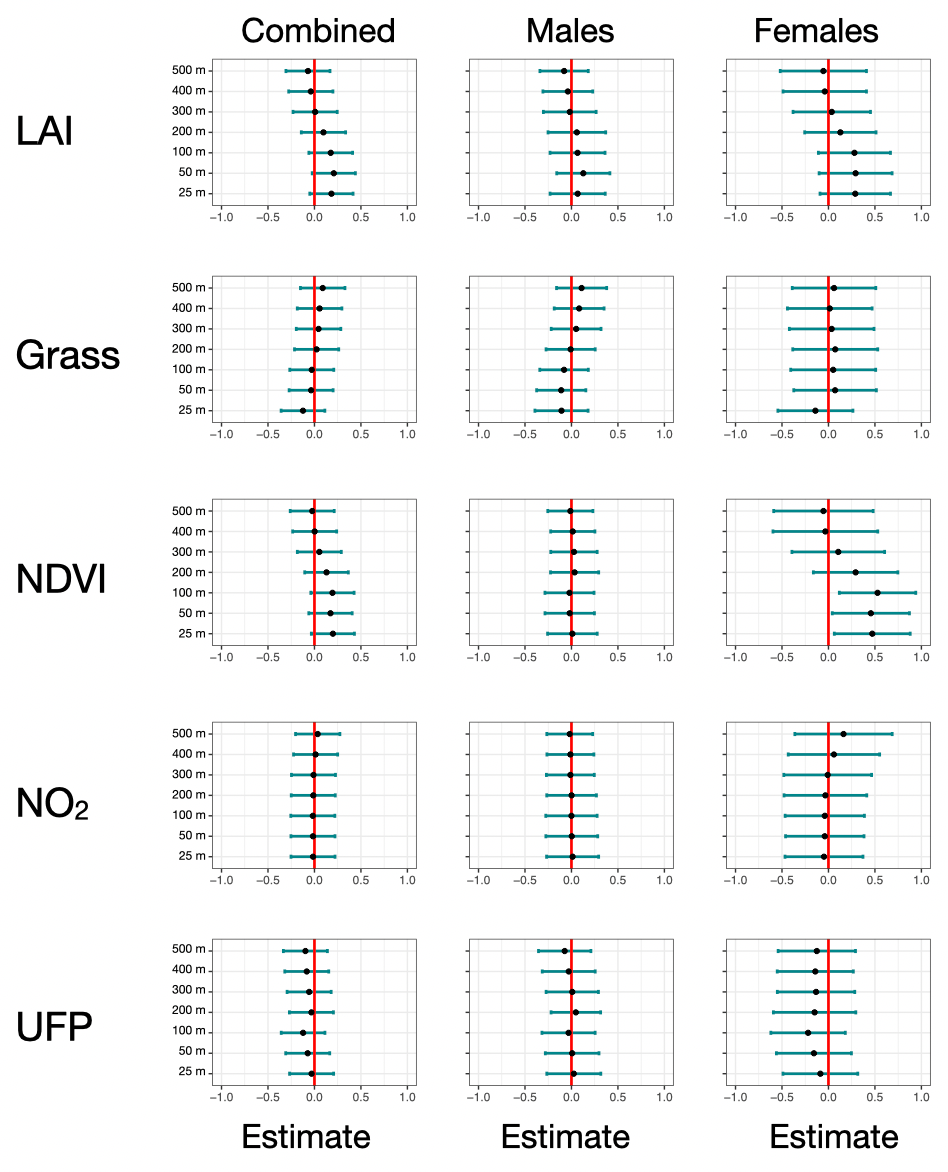
**
